# Coastal planktonic community unaffected by Boreal hydropower complex in Québec, Canada

**DOI:** 10.1007/s10661-021-09628-4

**Published:** 2022-01-05

**Authors:** Charles Deblois, Maud Demarty, Alain Tremblay

**Affiliations:** 1 Aqua-Consult, Montréal, QC Canada; 2 Direction – Environnement, Hydro-Québec, Montréal, QC Canada

**Keywords:** Hydropower, Estuary, Water temperature, Salinity, Chlorophyll *a*, Plankton

## Abstract

Comprehensive studies of the impact of hydropower on coastal environments are rare. This study examines the impact of commissioning the hydropower plants of the Romaine complex on the freshwater discharge of the Rivière Romaine near its estuary and on the Chenal de Mingan ecosystem in the summers of 2015, 2017 and 2019. Continuous temperature, salinity and chlorophyll *a* data were obtained from two instrumented buoys, and nutrients as well as the phytoplankton and zooplankton communities were sampled five times a year at 11 stations. The results demonstrate the major influence of offshore waters on temperature and salinity in the study area, and the decreasing influence of the Rivière Romaine with distance from its mouth. Nutrient concentrations in the estuary did not covary with river discharge or with nutrient concentrations in the river. Importantly, impoundment of the reservoirs of the complex had no measurable effect on nutrient stoichiometry in the Chenal de Mingan. Overall, the chlorophyll *a* concentrations ranged from 0.1 to 7.6 µg L^−1^ in the channel, the community was dominated by diatoms, and phytoplankton growth was either nitrate limited or under predation pressure. The zooplankton community has been composed of the same groups of species and has been dominated by cyclopoids and calanoids since 2015. Our study underlines the importance of including regional meteorological trends in the analysis to avoid biased conclusions on the impact of hydropower projects. The study concluded that modulation of the Rivière Romaine discharge and related changes in water quality did not lead to measurable change in plankton production in the Chenal de Mingan.

## Introduction

Freshwater is a natural resource essential for human activities such as agriculture, industry and energy generation and transmission. Global water demand for all uses, presently about 4 600 km^3^ per year, will increase by 20 to 30% by 2050 with the increase in human population and economic development (Burek et al., 2016; WWAP, 2019; Boretti, 2019). The persistent increase in reservoir construction for different purpose (flood control, irrigation, energy, etc.) is a notable indicator of this: in 1960, ten thousand large dams were listed; in 2020, sixty thousand were listed (ICOLD, 2020). Though large infrastructure projects are often controversial and can have negative environmental impacts (McAllister et al., 2001), engineers and stakeholder agree that an adaptative measure in current operation and creation of new reservoirs are necessary to offset the vulnerability of water resources systems to future climate uncertainties (Ehsani et al., 2017). The hydropower industry has not escaped the environmental debate, but it is still considered a major sustainable source of renewable energy (Moran et al., 2018).

The impact of reservoir creation on freshwater ecosystems and species at different latitudes is well documented, but studies of the downstream effects, on coastal areas in particular, are still rare (McAllister et al., 2001). Though the need for large-scale studies was underlined more than two decades ago (Rosenberg et al., 1997), dams are often constructed without adequate environmental impact assessments, especially of the effect of disturbed nutrient cycling on the downstream trophic chain. In north temperate or Boreal systems, hydroelectric developments trap high spring flows for storage in reservoirs and release higher-than-normal flows in winter when power is needed (Hydro-Québec, 2007; Métivier et al., 2018; Rosenberg et al., 1997). The changes in river flow as well the erosion (Schetagne & Verdon, 1999), sedimentation (Zang et al., 2016) and organic matter mobilization and remineralization (DeBonville et al. 2020) caused by dam creation can lead to drastic changes in nutrient exports from watershed to estuary and a severe decline in estuarine communities (McAllister et al., 2001; Maarava et al., 2020). In anthropogenically disturbed watersheds, on the other hand, dams act as nutrient and sediment sinks, which can have a positive effect on downstream estuarine water quality (Eccles et al., 2020). Even in the early years after first flooding, however, Canadian Boreal reservoirs and the river stretches downstream of them remain oligotrophic, like natural lakes in the region (Bogard & del Giorgio, 2016; Schetagne., 1994). As a result, dramatic positive or negative impacts are not anticipated. Nonetheless, with respect to the coastal trophic chain, discharge peaks, changes in seasonality of nutrient and organic matter exports from watershed to estuary and unbalanced nutrient stoichiometry at river mouths are concerns even in these cold environments (Senneville et al., 2018). Hence, when Hydro-Québec undertook to build a 1 550-MW hydropower complex consisting of four hydropower plants each with a reservoir on the Rivière Romaine in Boreal Québec, on the north shore of the Golfe du Saint-Laurent, the environmental impact study (EIS) underlined the need to document the project’s impacts on planktonic production in the river’s estuary and in the Chenal de Mingan. Thus, for the first time in Québec, a detailed long-term follow-up study was conducted to determine the drivers of plankton variation and to describe the plankton community itself.

Since freshwater from the Rivière Romaine accounts for at least half of the tidal prism in the Chenal de Mingan during the flood period and a fifth of it under low-flow conditions (Hydro-Quebec, 2007), the question was to know, whether the interannual variations in river discharge linked to commissioning and operation of the hydropower complex affected the plankton community. The growth of primary producers in the channel depends on multiple factors, including water temperature, salinity and concentrations of nutrients (nitrogen, phosphorus, silica, etc.) in the water column. The underlying hypothesis of this study was that modifications in river discharge would affect these parameters, possibly leading to a 5% increase in planktonic production in the study area and a shift in prevalent phytoplankton and zooplankton groups (Seneville et al., 2018). The EIS reports values for all the parameters studied. A supplementary study was conducted in 2013, before the first of the project’s four hydropower plants was commissioned, but for technical and logistical reasons, the methods used in this pre-flooding year differed from those used after flooding. The present study thus focuses mainly on interannual variations noted in the three study years following impoundment of the first of the four reservoirs of the Romaine complex (Romaine-2 was commissioned in 2014, Romaine-1 in 2015 and Romaine-3 in 2017, with Romaine-4 to become operational in 2022).

## Method

### Study area

**Fig. 1:**
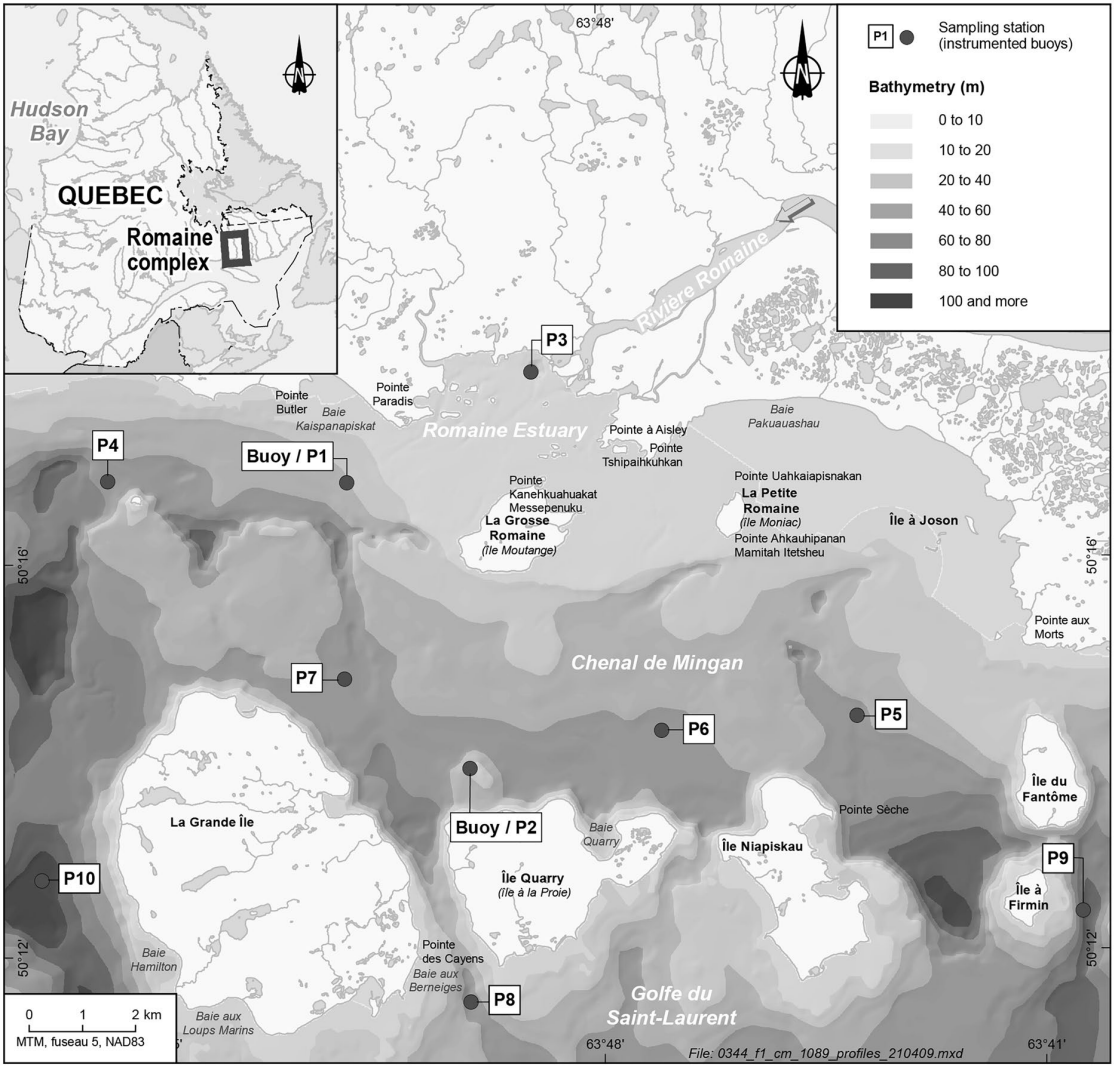
Study area, sampling stations (P0–P10) and buoy mooring sites (P1 and P2)

The study area covers a vast territory that extends into the Golfe du Saint-Laurent and the Archipel de Mingan, a chain of about 40 islands at the northern end of the Détroit de Jacques-Cartier in northern Québec, Canada (Fig. 1). It includes the mouth of the Rivière Romaine (estuary) and the western part of the Chenal de Mingan. This is a dynamic system influenced by strong tidal currents of high ecological and economical value. The Chenal de Mingan and the archipelago are east–west oriented and cover an area of about 170 km^2^. The mouth of the river consists in a shallow estuary covering about 14 km^2^ of sandy channels, banks and tidal flats plus large surface rocks brought in by ice. About 25% of this estuary is exposed at low tide. It sits on bedrock which outcrops at certain places in the Chenal de Mingan (Hydro-Québec, 2007). The river has one main outlet and two secondary outlets, where rock outcrops prevent the tide and salt wedge from migrating up the river. The water is 1 to 7 m deep in the estuary. In the Chenal de Mingan, the water averages about 50 m deep, ranging from 20 m to over 100 m deep (Lorrain et al., 2006).

### Hydrological regime of the river and estuary

The watershed of the Rivière Romaine covers 14,470 km^2^ (Hydro-Québec, 2007). It is a typical Boreal river, with a low discharge rate in winter, a spring flood at the onset of the watershed ice melt in May and June, and a moderate discharge rate in summer and autumn. Data from Hydro-Québec station ROMA0665, near the river mouth (KP 5.2), were used to calculate daily discharges under natural conditions (from 1999 to 2014) and for each study year (2013 and up). Minimum, maximum and daily discharge values were used to establish a reference hydrograph and envelope for the Rivière Romaine (1999 to 2014 data). The reference hydrograph was also used to identify three distinct hydrological periods, selected to facilitate interannual comparisons: historical flood period (HFP), summer-fall period (SFP) and glaciological-winter period (GWP). The HFP was defined by occurrence of daily flows greater than 500 m^3^ s^−1^, which appear on the reference hydrograph between May 3 and June 17. The absence of a systematic fall discharge peak led to a merging of the summer and fall flow data to create the summer-fall hydrological period (SFP). This period begins on June 18, immediately after the spring flood, and ends on November 11, 1 day before the onset of the glaciological winter. The GWP usually begins on November 12 and includes a few days of open water and then the entire period of ice cover of the estuary, ending immediately before the spring flood, on May 2.

The volume of the estuary’s tidal prism is about 20 hm^3^, for a mean tidal amplitude of 1.5 m over the estuary’s 14 km^2^. Freshwater volume delivered to the estuary by the Rivière Romaine in a half tidal cycle (approximately 6 h 10 min) is up to 32 hm^3^ at peak flow (1 500 m^3^ s^−1^) and then drops to 10 hm^3^ at a discharge of 500 m^3^ s^−1^ and eventually to 4.3 hm^3^ under low flow conditions (discharge ≤ 200 m^3^ s^−1^). In other words, at a discharge of 500 m^3^ s^−1^ or more (the established flood discharge threshold), freshwater accounts for about half the tidal prism, leading to dynamic thermohaline stratification patterns.

### Field sampling

Field data were collected in surveys complementary to those required under Hydro-Québec’s monitoring program. An intensive follow-up of physical, chemical and biological parameters in the Romaine estuary was conducted in 2013, 2015, 2017 and 2019. Though 2013 was considered the baseline year, the protocols and methods used for nutrient, phytoplankton and zooplankton analysis in that year were not designed to specifically answer the questions addressed here, and they differed from those used in 2015 and subsequent years. Hence, the 2013 dataset served only partially as a baseline for this study: only the temperature, salinity and chlorophyll *a* data gathered that year are fully comparable, given method continuity with subsequent years, and can illustrate the freshwater footprint under natural conditions.

Continuous and spatial measurements were taken from spring to autumn in 2013, 2015, 2017 and 2019. Continuous data were obtained from two instrumented buoys deployed every year in the Chenal de Mingan before the HFP in early spring and retrieved in mid-September during the SFP. The first buoy was moored about 2.8 km downstream of the river mouth, just outside the main estuary channel (P1, “littoral”, Fig. 1) and the second buoy about 7.7 km from the river mouth, within the zone protected by the archipelago (P2, “offshore”, Fig. 1). Both buoys were equipped with a multiparameter (conductivity, temperature and depth, CTD) logger (RBR) and a fluorometer (Seapoint) for chlorophyll *a* measurement. The sensors were positioned at a depth of 1.0 m and measured sub-surface variations in the targeted parameters every 10 min. The offshore buoy also included a quantum sensor (Biospherical QSR-2200) and a weather station (Weatherpack 2000). All data were validated using an automated process to eliminate outliers and data outside probe specifications. Data from the quantum sensor were used to define days and nights to validate the fluorometer data, since laboratory testing and collected field data showed clear evidence of quenching under direct sunlight. To minimize this effect, all fluorometer data recorded in the presence of light were removed from the final dataset. The minimum and maximum tidal values reported by Environment Canada at Havre-Saint-Pierre (QC, Canada) were used to calculate water level in the study area at each interval and thus determine tidal stage for each record. The water level data were presented on a uniform time vector with an interval of 10 min and were corrected by a time factor of 27 min to account for the 10-km distance between Havre-Saint-Pierre and the study site.

Spatial data were obtained from discrete water samples taken at 11 stations (P0 to P10, Fig. 1) in five field campaigns between the HFP and SFP of each study year. The stations were strategically positioned to cover the freshwater gradient of the study area, with P0 directly in the river and representing the river signature, P3 positioned near the river mouth and the other stations covering the Chenal de Mingan (P1, P2, P4, P5, P6 and P7) and the southern part of the archipelago in the Détroit de Jacques-Cartier (P8, P9 and P10). At each station, water samples were collected in triplicate using a tubular integrator (Majaneva et al., 2009) or a Niskin bottle (P0 only). The integrator was deployed in the upper photic zone, i.e. the depth at which light intensity (calculated from direct underwater PAR measurements obtained in situ with a LI-COR LI-250A light meter coupled with a LI-COR LI-193 spherical quantum sensor and a LI-COR LI-190 surface PAR sensor) drops to 10% surface irradiance. Integration depth averaged 7.6 m over the surveys, ranging from 0.5 to 16 m, with low values mostly encountered at P3. Nutrients and chlorophyll *a* were collected from the integrated samples, a total of 165 samples per year. Each nutrient sample was filtered directly after collection with a clean syringe on a polyethersulfone membrane (Millipore, 0.22 µm) and stored in the dark at − 20 °C until analysis for nitrites and nitrates (TDN), total dissolved phosphorous (TDP) and dissolved silicate (TDSi) as per Armstrong et al. (1967) and Grasshoff et al. (1983). Chlorophyll *a* samples were kept cold in the dark on the boat and then filtered through a 47-mm fibreglass filter, 0.7 μm pore size (Millipore, GFF), in a dark room at the end of each sampling day, the filters then immediately stored at − 20 °C until extraction and analysis according to Trees et al. (2002). Phytoplankton samples were collected from the first integrated triplicates at each station (total of 55 samples per year), immediately fixed with acidic Lugol’s solution at a ratio of 1 mL per 250 mL of sample and then stored in amber glass bottles at ambient temperature until taxonomic composition analysis with an inverted microscope according to Planas et al. (2000). Phytoplankton biovolume was estimated from direct biometric measurements of cells using conversion factors in the literature (Hillebrand et al., 1999).

Zooplankton samples were collected at stations P1 to P7 using a ring net (3 m long, 0.75 m in diameter, 150 µm mesh size) equipped with a one-way clutch flowmeter (General Oceanic). The net was vertically pulled at constant speed to collect an integrated representative sample of organisms in the water column, from bottom to surface. Upon retrieval, the zooplankton samples were concentrated using a 150-µm filter, anesthetized with 50 mL of sparkling water, fixed with two volumes of 95% ETOH per volume of zooplankton and stored at 4 °C for 48 h. Each sample was then gently rinsed on a 150-µm mesh filter with 95% ETOH and the organisms were stored in fresh 95% ETOH until counting and identification according to Ward (1955) and Gannon (1971). Organism abundance was calculated using the water volume filtered, which was estimated owing to the flowmeter calibration curve.

### Chlorophyll *a* data

The fluorometer data from the instrumented buoys was validated in 2015, 2017 and 2019 by water sampling at instrument depth during each sampling campaign. A total of 15 comparison points were obtained over the years at P1 and P2. Laboratory analyses returned concentrations ranging from 0.3 to 3.24 µg L^−1^ with an average of 0.99 µg L^−1^ (SD ± 0.7), while the fluorometers on the buoys recorded concentrations between 0.2 and 2.25 µg L^−1^ with an average of 0.98 µg L^−1^ (SD ± 0.5). The means comparison was not significant for the data gathered at P1, P2 and altogether (Student paired test, *p* = 0.91). Chlorophyll *a* concentrations from the laboratory analyses were therefore used for most of the analyses described below. The fluorometer time series, however, was considered robust enough to interpret dynamic variation in the study area between sampling campaigns.

### Plankton analysis

Once counted and identified, the phytoplankton were concatenated into six functional groups (brown algae, green algae, blue-green algae, diatoms, flagellates and other) to identify major shifts in population composition. A preliminary analysis of group distribution and abundance showed some stations presented a unique pattern while others were similar to one another. Accordingly, stations P0, P1 and P3 (along the axis of the Rivière Romaine and the estuary) were analyzed individually while the western stations (P2, P4 and P7) and the eastern stations (P5 and P6) as well as those representing the phytoplankton signature off the Archipel de Mingan (P8, P9 and P10) were all grouped together. The same approach was taken to analyze the zooplankton community. The organisms identified were classified into six groups, i.e. cladocerans, calanoids, cyclopoids, harpacticoids, nauplii and other. Preliminary comparisons showed quite similar community covariation in stations P1, P2, P4, P5, P6 and P7 while the data from station P3, at the river mouth, was different from one year to the next. The zooplankton community specific to P3 was thus analyzed separately.

## Results

### Hydrological characteristics of the river

**Fig. 2:**
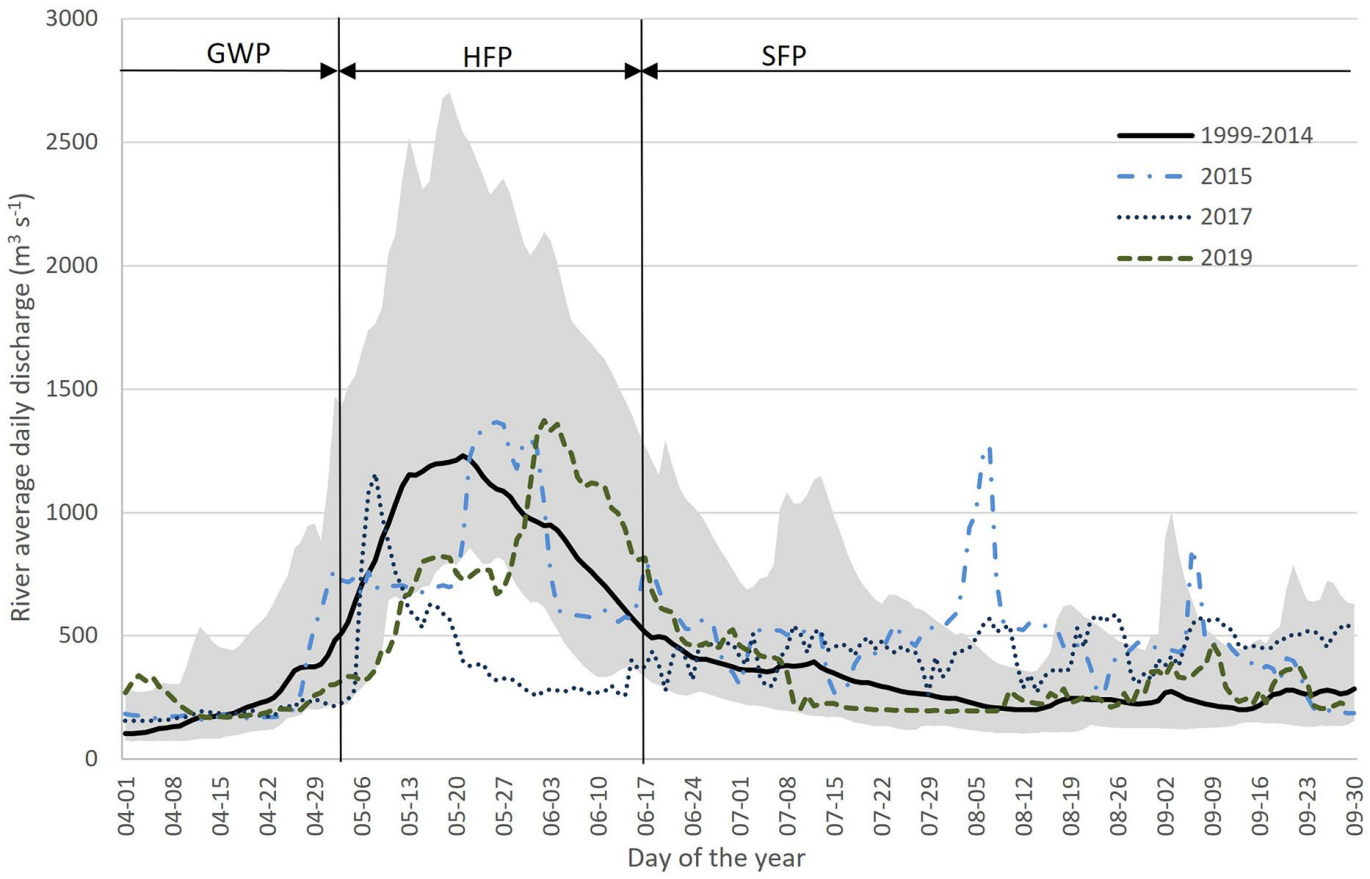
Rivière Romaine average daily discharge (m^3^ s^−1^) for study periods. GWP for glaciological-winter period, HFP for historical flood period, SFP for summer fall period. The grey area represents the range (min to max) of daily discharge measured during the reference period (1999–2014)

Hydrographs of the Rivière Romaine between April 1 and September 30 (the study period) in each study year were compared using both the average flow and the seasonal pattern obtained for the study period (Fig. 2 and Table 1). Historically (1999–2014), average flow rate in the Rivière Romaine during the study period was 436 m^3^ s^−1^ (SD ± 314) with a typical peak freshet flow of 1230 m^3^ s^−1^ usually occurring on May 21. In 2013, the average discharge was not significantly higher, at 450 m^3^ s^−1^ (SD ± 379; ANOVA, *p* > 0.05). The first year after commissioning of the Romaine-2 generating station (2015), the river discharge exceeded the historical freshet threshold of 500 m^3^ s^−1^ throughout the study period, averaging 526 m^3^ s^−1^ (SD ± 283; highest rate for the analyzed dataset; ANOVA, *p* < 0.05), with flow rates high during the HFP, but also during the SFP. This was partly due to the artificial discharge modulation required to build the Romaine-1 development and abundant precipitation throughout the watershed over the summer of 2015. In 2017, the discharge was modulated not only by operation of Romaine-2 and Romaine-1 (commissioned in late 2015) but also by first filling of the Romaine-3 reservoir. These conditions led to a decrease in average discharge over the study period (401 m^3^ s^−1^ (SD ± 166), the hydrograph showing a particularly weak freshet period, with high flow (> 500 m^3^ s^−1^) occurring on only 14 of the 46 expected HFP days; however, steady high flows experienced during summer led to an average discharge statistically comparable to the reference period (Tukey test). Unlike in previous years, operating conditions in 2019 resulted in a hydrograph with trends very similar to those of the reference period (1999–2014; no significant difference according to Tukey’s test). Average discharge was 418 m^3^ s^−1^ (SD ± 295) and there were 38 days of spring freshet conditions followed by a sustained summer low flow rate.

**Table 1:** Daily average flow rates measured at KP 5.2 of the Rivière Romaine and total exported volume of water between April 1 and September 30 in the historical period (1999–2014) and the study years (2013, 2015, 2017 and 2019)

Parameter	Historic (1999–2014)		2013		2015		2017		2019	
	Average	SD/date	Average	SD/date	Average	SD/date	Average	SD/date	Average	SD/date
01/04–30/09	436	± 314	450	± 379	526	± 283	401	± 166	418	± 295
HFP	922	± 227	1010	± 337	826	± 278	441	± 238	826	± 300
HFP (peak and date)	1230	May 21	1610	May 12	1366	May 26	1158	May 9	1374	June 2
SFP	287	± 76	272	± 113	482	± 179	448	± 80	298	± 114
Exported volume (hm^3^)	Sum	Diff. (%)	Sum	Diff. (%)	Sum	Diff. (%)	Sum	Diff. (%)	Sum	Diff. (%)
	6274	Not applicable	6482	3.3%	7658	22.1%	5817	− 7.3%	5985	− 4.6%

Historical average flow rate during the HFP (May 3 to June 17) was 922 m^3^ s^−1^ (SD ± 227). In 2013, the HFP average was higher, 1 010 m^3^ s^−1^ (SD ± 337), with peak flow reaching 1 610 m^3^ s^−1^, the 90th percentile for historically measured maximum flow rates. Average HFP flow rates were identical in 2015 and 2019, and at 826 m^3^ s^−1^ not significantly different from the historical rate (SD ± 278 for 2015 and SD ± 300 for 2019; Dunnett test, *p* > 0.05). The first filling of the Romaine-3 reservoir in 2017 resulted in an exceptionally low HFP average flow rate of 441 m^3^ s^−1^ (SD ± 238). Interestingly, despite these interannual differences in HFP average flow rates, HFP peak values were similar in all study years (1 230, 1 366, 1 158 and 1 374 m^3^ s^−1^ for 1999–2014, 2015, 2017 and 2019, respectively, Table 1). Because of the high average discharge and the high peak flow in 2013, the freshet that year was exceptionally strong compared to historical conditions.

The SFP data collected also reflect flow rates variability following commissioning of the Romaine complex hydropower plants. The historical average flow rate between June 18 and September 30 was 287 m^3^ s^−1^ (SD ± 76), i.e. about 31% of the average freshet discharge. In 2013, it was 272 m^3^ s^−1^ (SD ± 112) or 27% of the average freshet. In 2015 and 2017, after commissioning of two of the Romaine complex plants, the SFP river discharge was significantly higher, at 482 m^3^ s^−1^ (SD ± 179) and 448 m^3^ s^−1^ (SD ± 80), i.e. 58% and 102% of the average freshet discharge respectively (ANOVA, *p* < 0.05 and Tukey test). In 2019, after three plants had been commissioned, however, it was representative of historical conditions at 298 m^3^ s^−1^ (SD ± 114), i.e. 36% of the average freshet discharge.

To understand the effect of interannual variations, the discharge data were also analyzed using a volumetric approach: historically, 6 274 hm^3^ of water was exported annually at the river mouth between April 1 and September 30. With the high average discharge in 2015, the amount of water exported was significantly higher, 7 658 hm^3^, or a net increase of 22.1% (ANOVA, *p* < 0.05; with Tukey’s test showing that 2015 is the only year significantly different). Partly due to modulation of the discharge for construction of the Romaine-1 development, this increase generated unanticipated but interesting conditions in the estuary, allowing measurement of the potential impact of a sustained freshwater plume in the study area throughout the study period. In 2017, freshwater exports were the lowest recorded during the HFP, but this was mitigated by a high and steady discharge during the SFP. The combination of these conditions resulted in a net export loss of only 7.3% (5 817 hm^3^) compared to 1999–2014 conditions. In 2019, the discharge was not significantly different from historical conditions during both the HFP and the SFP, resulting in a total net freshwater export loss of 4.3% (5 985 hm^3^). In fact, the 2019 hydrograph matches the anticipated restitution pattern under normal operating conditions according to the project’s EIS (Hydro-Québec, 2007). In other words, the 2019 hydrograph and the resulting decrease in net freshwater exports is likely indicative of conditions to be expected at the river mouth in the future.

### Temporal trends in temperature and salinity

**Fig. 3:**
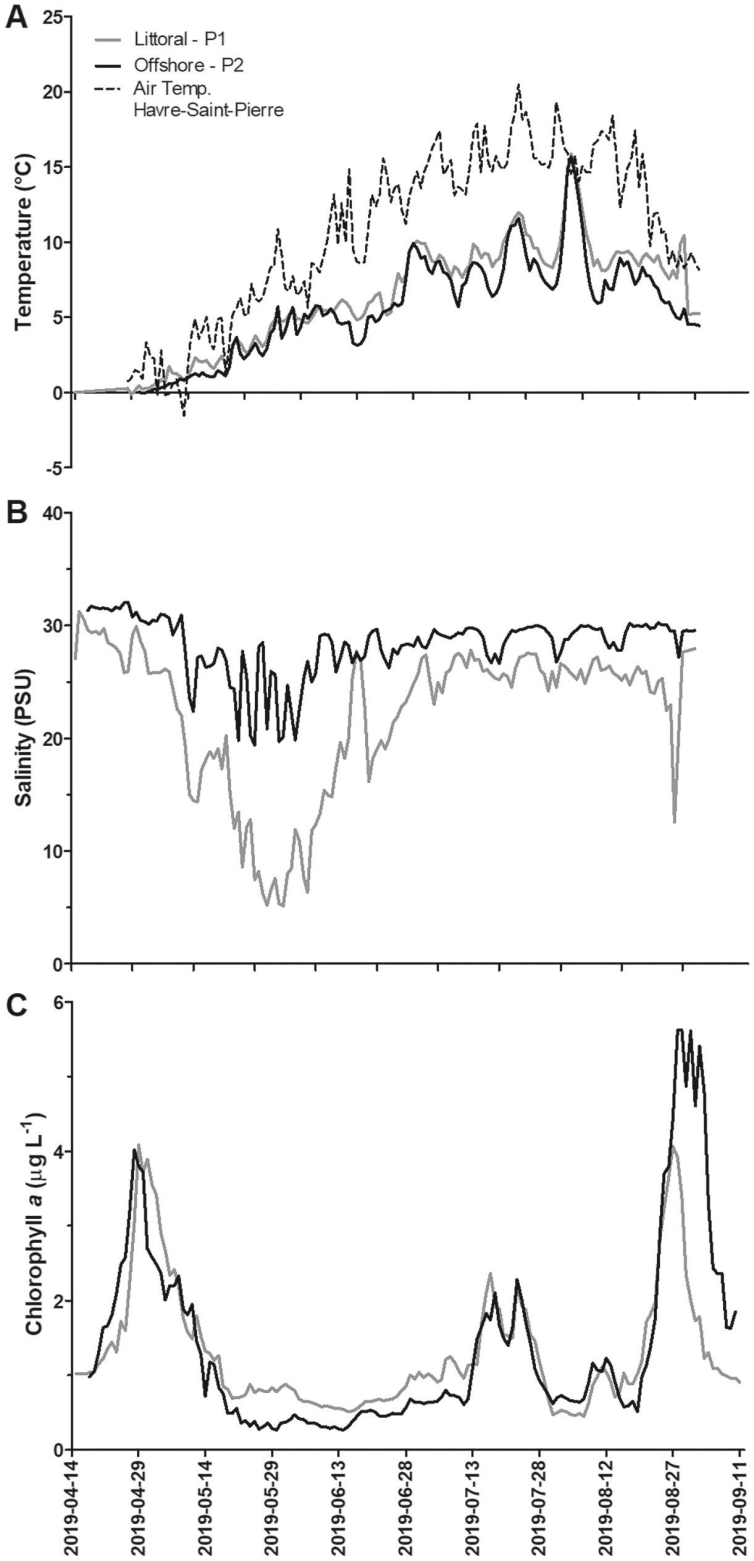
Temperature (**A**), salinity (**B**) and chlorophyll *a* (**C**) time series obtained in 2019 at littoral (P1) and offshore (P2) stations

Surface water temperatures measured by the instrumented buoys as well as air temperatures recorded at Havre-Saint-Pierre weather station were linked to the seasonal cycle. As expected, the air and the water gradually warmed up as the ice cleared out and over the study period. The temperature data collected in 2019 and associated variation are typical of earlier years and are used here to describe the system. Air temperatures rise from close to 0 °C in April to a maximum of 25 °C in August and show an average temperature of 10.7 °C (± 5.4) for the study period as a whole. As for surface water temperature, it was slightly below freezing point in mid-April and increased to a maximum of 17.5 °C at P1 and 18.7 °C at P2 at the end of July and the beginning of August and then dropped continuously until the end of the study period (Fig. 3). Except on rare occasions, surface water temperature was warmer at P1 than P2 every study year. In 2019, the difference between the stations was on average 0.8 °C, comparable to the differences observed in 2013 (1.1 °C), 2015 (0.9 °C) and 2017 (1.5 °C). As expected, this difference is related to the positions of the buoys with respect to the river mouth and the colder saline water from the Détroit de Jacques-Cartier. Station P1 is near the estuary, in shallow water, and closer to potentially warmer freshwater input from the river. Station P2 is in the Chenal de Mingan, which is influenced by the cold saline water from the Détroit de Jacques-Cartier every tidal cycle.

Salinity also differed between P1 and P2. Since the beginning of the monitoring in 2013, salinity was lower at P1, with an overall average of 19 PSU at P1 during the HFP compared to 27 PSU at P2. The difference was less during the SFP, with 25 PSU at P1 compared to 29 PSU at P2. As expected, surface salinity also oscillated daily and the range of variation covaried in function of (1) the interplay between tide forcing occurring twice a day, (2) freshwater exports during the study period and (3) distance from the river mouth. These factors interacted and modified the relative volumes of fresh and saline water in the intertidal prism. This modulated the surface salinity measured by the instrumented buoys and created oscillations in synchronization with the tidal cycle. The combined effect of low tide and a high discharge rate occasionally leads to null values in surface salinity at both stations. This was observed every year at P1 (frequently) and at P2 (mostly during the freshet). Conversely, high tide combined with a low discharge rate place station P2 outside the influence of the intertidal prism, and subsurface instruments record euryhaline conditions. This analysis was supported by an ANCOVA analysis, which demonstrated an inverse correlation between salinity and discharge from the Rivière Romaine from 2013 to 2019, with the relationship stronger at P1 (regression, *R*^2^ = 0.69) than at P2 (regression, *R*^2^ = 0.47).

### Nutrients

Total dissolved nitrogen (TDN) and chlorophyll *a* concentrations were analyzed along the freshwater gradient, the results from P0 representing inputs from the Rivière Romaine, those from P3 representing the estuary area and those from the nine other sampling stations representing the Chenal de Mingan. TDN was specifically chosen for analysis because it is a key element in controlling phytoplankton growth in coastal systems (Blais et al., 2019; Howarth & Marino, 2006).

Concentrations of TDN varied over the study period (April 1 to September 30). Before any of the hydropower plants of the Romaine complex were commissioned, TDN concentrations in the Rivière Romaine were higher in winter and spring (50 to 140 µg L^−1^) and lower in summer (10 to 20 µg L^−1^) (Hydro-Québec, 2007). These seasonal TDN trends remained after the sequential commissionings: in 2015, 2017 and 2019, TDN concentrations measured at P0 were generally higher in April and/or May (overall concentration 38 µg L^−1^, SD ± 15; ANOVA, *p* > 0.05), and decreased in July, August and September (average 29 µg L^−1^, SD ± 8, Table 2). Overall, average concentrations varied significantly from one year to the next: 23 µg L^−1^ in 2015, 42 µg L^−1^ in 2017 and 33 µg L^−1^ in 2019 (ANOVA, *p* < 0.05). In general, TDN concentrations measured at the mouth of the river (station P3) are between those observed in the river (P0) and those measured in the Chenal de Mingan. Accordingly, concentrations were 35 to 51 µg L^−1^ during the HFP (April and May) when the influence of the river is greater, and then decreased during the SFP to 17 to 32 µg L^−1^, sometimes with a slight uptick in September. In the Chenal de Mingan, average TDN concentrations were high in April, before the HFP, and then tended to decrease until July (Table 2). The trend for subsequent months was variable: in 2019 an increase was noted in August and September, while in 2017 nitrate concentration increased in August but declined in September. Concentrations measured at the different stations over the years ranged from 4 to 135 µg L^−1^, and there were no differences in averages calculated for the different Chenal de Mingan stations (Tukey test, *p* > 0.05), demonstrating that TDN concentrations are homogenous outside the Romaine estuary. Globally, average TDN concentrations in the Chenal de Mingan were 46 µg L^−1^ (SD ± 29), 34 µg L^−1^ (SD ± 12) and 45 µg L^−1^ (SD ± 35) in 2015, 2017 and 2019, respectively, i.e. significantly higher than in the Rivière Romaine for 2015 and 2017 (Student’s *t*-test, *p* < 0.05).

**Table 2:** Nutrients and chlorophyll *a* concentrations and stoichiometric ratios calculated for different years and sampling periods

Year of sampling	Campaign	Month	Study area	Total dissolved nitrogen µgN L^−1^		Total dissolved phosphorous µgP L^−1^		Total dissolved silica µgSi L^−1^		N/P ratio		Si/N ratio		Chlorophyll *a* µg L^−1^	
				Mean	SD	Mean	SD	Mean	SD	Mean	SD	Mean	SD	Mean	SD
2015	C1	April	Romaine River	10	1	1	0	163	17	8.5	0.2	17.4	3.6	na	na
			River mouth	na	na	na	na	na	na	na	na	na	na	0.9	0.1
			Mingan Channel	94	14	26	2	192	36	3.6	0.3	2.0	0.2	1.3	0.3
	C2	May	Romaine River	35	0	4	0	42	3	8.4	0.4	1.2	0.1	0.4	0.0
			River mouth	35	0	5	0	43	1	7.3	0.6	1.2	0.0	1.0	0.0
			Mingan Channel	54	10	21	3	157	118	2.6	0.3	2.9	2.2	1.5	0.7
	C3	June/July	Romaine River	25	1	1	0	44	1	18.2	1.2	1.8	0.0	0.9	0.0
			River mouth	23	2	1	0	41	2	21.0	0.2	1.7	0.1	0.5	0.1
			Mingan Channel	51	11	20	2	203	112	2.6	0.4	4.1	2.1	0.9	0.8
	C4	August	Romaine River	24	1	0	0	65	11	49.6	1.1	3.1	*	0.6	0.0
			River mouth	21	1	0	0	69	4	204.3	43.7	3.2	0.2	0.8	0.2
			Mingan Channel	14	4	13	1	175	87	1.1	0.3	13.0	6.7	0.8	0.3
	C5	September	Romaine River	22	1	1	0	36	7	17.0	0.9	1.7	0.2	0.5	0.0
			River mouth	19	0	0	0	39	9	51.5	15.0	2.0	0.4	0.6	0.0
			Mingan Channel	27	13	15	3	188	134	1.7	0.7	6.9	2.8	0.9	0.2
2017	C1	April	Romaine River	51	0	2	0	90	16	22.8	2.4	1.8	0.3	0.3	0.0
			River mouth	48	1	2	0	228	74	19.9	2.8	4.8	1.5	0.3	0.0
			Mingan Channel	37	10	18	1	120	35	2.0	0.4	3.3	0.8	3.0	0.6
	C2	May	Romaine River	48	4	5	1	1	0	9.3	1.2	0.0	0.0	0.2	0.0
			River mouth	50	1	5	0	25	7	9.3	0.1	0.5	0.1	0.2	0.0
			Mingan Channel	16	9	12	4	186	155	2.1	2.6	10.2	4.4	0.9	0.4
	C3	June/July	Romaine River	42	15	2	1	64	6	17.8	2.9	1.6	0.4	1.0	0.0
			River mouth	32	0	1	0	51	8	26.2	7.9	1.6	0.3	0.5	0.0
			Mingan Channel	41	7	17	2	143	82	2.4	0.2	3.7	2.8	0.6	0.1
	C4	August	Romaine River	38	4	1	0	46	5	30.0	6.7	1.2	0.1	0.4	0.0
			River mouth	27	0	2	0	141	15	16.5	0.7	5.1	0.5	0.3	0.0
			Mingan Channel	45	7	18	1	127	26	2.5	0.4	2.9	0.8	0.4	0.1
	C5	September	Romaine River	28	3	1	1	156	83	23.7	10.8	5.3	2.4	0.8	0.1
			River mouth	23	1	2	0	201	16	11.4	0.9	8.8	0.9	1.0	0.2
			Mingan Channel	31	3	18	1	150	42	1.8	0.2	4.8	1.3	0.5	0.1
2019	C1	April	Romaine River	43	0	2	0	152	1	21.2	0.7	3.5	0.0	0.4	0.1
			River mouth	49	1	7	0	386	22	7.0	0.2	7.8	0.3	0.3	0.0
			Mingan Channel	111	11	27	3	287	33	4.1	0.3	2.6	0.4	0.6	0.1
	C2	May	Romaine River	47	*	2	*	320	*	29.7	*	6.8	*	0.3	0.0
			River mouth	36	1	2	0	110	1	16.9	0.5	3.1	0.1	0.4	0.0
			Mingan Channel	25	10	13	2	97	75	1.9	0.7	3.6	2.0	2.0	0.4
	C3	June/July	Romaine River	23	0	1	0	166	2	22.2	0.4	7.2	0.1	0.7	0.1
			River mouth	19	0	3	0	352	4	6.0	0.2	18.6	0.4	0.4	0.0
			Mingan Channel	15	6	12	3	72	42	1.3	0.3	4.9	3.5	1.8	1.1
	C4	August	Romaine River	30	0	2	0	171	1	20.2	0.3	5.7	0.0	0.4	0.0
			River mouth	18	0	2	0	642	5	8.4	0.5	36.7	0.8	0.4	0.0
			Mingan Channel	28	5	13	1	137	16	2.0	0.2	5.0	0.8	0.6	0.2
	C5	September	Romaine River	28	0	2	0	194	*	11.7	0.5	6.9	*	0.8	0.2
			River mouth	26	1	2	0	656	0	11.5	0.6	25.2	0.6	0.7	0.2
			Mingan Channel	46	3	17	1	221	73	2.7	0.2	4.8	1.6	1.2	0.3

^*^n = 1

*Na*, not available

In August and September 2017, exploratory measurements of dissolved ammonium concentrations were taken. Values were systematically lower than 1 µg L^−1^ in the Rivière Romaine, confirmed in the systematic sampling program in 2019. Concentrations measured in the Chenal de Mingan, on the other hand, ranged from 27 to 64 µg L^−1^ in 2017, i.e. in the same range as nitrate concentrations. In 2019, dissolved ammonium concentrations gradually decreased over the summer, from 35 µg L^−1^ in April to 10 µg L^−1^ in September.

Total dissolved phosphorus (TDP: P-PO_4_) concentrations in the waters of the Rivière Romaine remained low (< 2.5 µg L^−1^) in all campaigns in 2015 and 2017, except during the spring freshet when a temporary increase was observed (4.5 µg L^−1^ and 5.2 µg L^−1^, respectively, Table 2). TDP concentrations in the river had a lower range in 2019, varying between 1.0 and 2.4 µg L^−1^, and remained stable throughout the study period, averaging 1.7 µg L^−1^. The concentrations in the river did not vary significantly between sampling years (ANOVA, *p* > 0.05). In the Chenal de Mingan, TDP concentrations ranged from 12.7 to 25.9 µg L^−1^ . They were consistent across all sampling stations and higher in April (mean 25.9 µg L^−1^; (ANOVA, *p* < 0.05)) than during the other sampling periods. There was a strong linear relationship between phosphorus and nitrate concentrations (*R*^2^ = 0.79). Like TDN, at the river mouth (station P3), phosphorus concentrations were in between those in the Chenal de Mingan and the Rivière Romaine, ranging from 0.02 to 11.8 µg L^−1^, but varied like those of the Chenal de Mingan even though diluted by freshwater input.

Dissolved silica (Si-SIO_4_; reactive silica) concentrations measured in the Rivière Romaine (station P0) since 2015 ranged from 1.0 to 320 µg L^−1^ (Table 2). There was no seasonal pattern nor any relation to river discharge, since both minimum and maximum concentrations were recorded during the freshet (in 2017 and 2019, respectively). Average concentration was similar in 2015 and 2017, approximately 70 µg L^−1^, but was significantly higher in 2019 at 182 µg L^−1^. At the sampling stations in the Chenal de Mingan, silica concentrations ranged from 4 to 508 µg L^−1^. Concentrations measured in 2015 were significantly higher than the other years, but there was no evidence of a seasonal or river discharge effect. Interestingly, the highest average concentrations in the Chenal de Mingan (all years and campaigns included) were noted at the stations closest to the shore, P1 and P4 (ANOVA, *p* < 0.05), where concentrations of 286 µg L^−1^ (SD ± 112) and 211 µg L^−1^ (SD ± 110), respectively, were recorded, comparable to the average concentration of 221 µg L^−1^ (SD ± 110) noted at the river mouth (station P3). Average concentration at the other stations in the Chenal de Mingan was lower, at 139 µg L^−1^ (SD ± 73). There was no relationship between silica and phosphorous concentrations and only a weak correlation with nitrogen (*R*^2^ = 0.37). Unlike with the other nutrients, silica concentrations in 2019 at the river mouth (station P3) were not in between those in the river and those in the Chenal de Mingan, ranging overall from 20 to 221 µg L^−1^. Local conditions in the estuary apparently tend to favour silicate release from sediment, but the silicate dispersion was limited to the estuary and did not modify silicate content in the Chenal de Mingan.

In the Rivière Romaine, average stoichiometric N:P ratios (molecular weight basis) ranged from 8.4 to 49.6, with very similar annual averages: 20.0, 20.7 and 19.4 in 2015, 2017 and 2019, respectively (ANOVA, *p* > 0.05). In the Chenal de Mingan, N:P ratios were ten times lower, ranging from 1.1 to 4.1. As in the river, annual averages were similar: 2.2, 2.2 and 2.4 in 2015, 2017 and 2019, respectively (ANOVA, *p* > 0.05). Ammonium measurements in 2019 showed ammonium to be sufficiently abundant to alleviate the nitrogen limitation in the study area. NH_4_ concentrations were in fact of the same order of magnitude as nitrate concentrations. Taking NH_4_ into account changes the availability of nitrogen in the water column, increasing the N:P ratio from 2.4 to 3.7. The Si:N stoichiometric ratio in the Rivière Romaine was more variable, ranging from 0.02 to 17.3, but with similar annual averages of 5.2, 1.9 and 5.6 in 2015, 2017 and 2019, respectively (ANOVA, *p* > 0.05). In the Chenal de Mingan, the Si:N ratio ranged from 2.0 to 13.0, with an average of 5.0 (Table 2, no significant differences; ANOVA, *p* > 0.05). Local release of silicate at the river mouth generated a higher Si:N ratio there in 2019, with an average of 18.3, but there were no differences between the river mouth and the Chenal de Mingan in earlier years.

### Chlorophyll *a*

Chlorophyll *a* concentrations in the Rivière Romaine (station P0) were very low in all study years and in all seasons, ranging from 0.2 to 1.0 µg L^−1^ with an average of 0.5 µg L^−1^ (SD ± 0.2) and no significant differences between years (ANOVA, *p* > 0.05). Seasonal data show the lowest chlorophyll *a* level at this station was recorded in May (0.3 µg L^−1^) and the highest in late June/early July (0.9 µg L^−1^) (ANOVA, *p* < 0.05). Chlorophyll *a* concentrations at the river mouth (station P3) were similar to those recorded in the river, with values ranging from 0.2 to 1.2 µg L^−1^ and an overall average of 0.5 µg L^−1^ (SD ± 0.3), but here there were significant differences between years, the average chlorophyll *a* concentration being higher in 2015 than in 2017 and 2019 (0.8, 0.4 and 0.4 µg L^−1^, respectively, ANOVA, *p* < 0.05) with no differences noted between campaigns (ANOVA, *p* > 0.05). In the Chenal de Mingan, chlorophyll *a* concentrations were generally higher, ranging from 0.2 to 4.6 µg L^−1^ with an average of 1.1 µg L^−1^ (SD ± 0.8), and discrete water samples showed no differences in concentrations among study years or most stations. The highest values were recorded at station P9, such that the overall average at this station (still oligotrophic at 1.5 µg L^−1^) was significantly higher than the averages for the other stations in the Chenal de Mingan (ANOVA, *p* < 0.05).

Chlorophyll *a* concentrations were also estimated by the fluorometer on the instrumented buoys at 10-min intervals for the entire duration of the study. Time series (Fig. 3 for 2019) show that spring peaks of chlorophyll *a* occurred between April and the onset of the freshet (HFP) in every study year. These peaks occurred between the field campaigns, underlining the utility of autonomous systems, such as instrumented buoys. The data also show that the magnitude of the chlorophyll *a* peak varies from one year to the next. From the beginning of the surveys to the spring peaks occurrence, all years included, the daily averages of chlorophyll *a* concentration ranged from 0.5 to 4.1 µg L^−1^ at P1 and from 1.0 to 4.4 µg L^−1^ at P2, with maximum values obtained on April 9, 2019, and May 2, 2015, respectively. Every year, chlorophyll *a* declined during or shortly after the spring freshet, when minimum values (between 0.1 and 0.3 ug L^−1^) were observed at both stations. A cyclic small flowering (± 2 µg L^−1^) occurred every year in July, followed by a larger flowering event (4.1 to 7.6 µg L^−1^) at the end of the month of August, as seen in 2017 and 2019. No relationship was found between chlorophyll *a* concentrations (time series or discrete sampling) and river flow rate, surface water temperature or nutrients (linear regression and multivariate analysis tested, all unsignificant with *p* > 0.05).

### Phytoplankton dynamics

A total of 230, 210 and 237 species of phytoplankton from 29, 26 and 28 classes of organisms were identified in samples taken in the photosynthetically active zone of the Chenal de Mingan and in the Rivière Romaine in 2015, 2017 and 2019 respectively. This great diversity of organisms included representatives of several major phytoplankton groups, among them chlorophytes, cyanophytes and bacillariophytes. The detailed taxonomic analysis is not included here. The dominant classes are briefly presented below according to abundance, with details on biovolume.

In spring (April and May), picoplankton cyanobacteria dominated the phytoplankton community in terms of abundance, accounting for up to 98% of counted cells (station P6, May 2017). In June/July, the community was more variable depending on the year. In 2015, cyanobacteria dominated, with an average 85% of counted cells, and diatoms were poorly represented (3%). In 2017, one pattern was observed at the freshwater stations (station P0 in the Rivière Romaine and station P3 in the river mouth) and another at the other nine stations. At the freshwater stations, cyanobacteria dominated with 83% of the population, while at the other nine stations there was a shift to community dominated by diatoms, which accounted for 56% of the community. In 2019, this difference was further accentuated, with cyanobacteria accounting for 72% of the community at stations P0 and P3, but only 25% at the stations farther from the shore, where diatoms dominated with an average 63% of the community. The shift was short-lived, however, with an increase in cyanobacteria abundance offshore in August and September, and overall cyanobacteria abundance ranging from 61% in September 2019 to 84% in August 2015 and 2017.

**Fig. 4:**
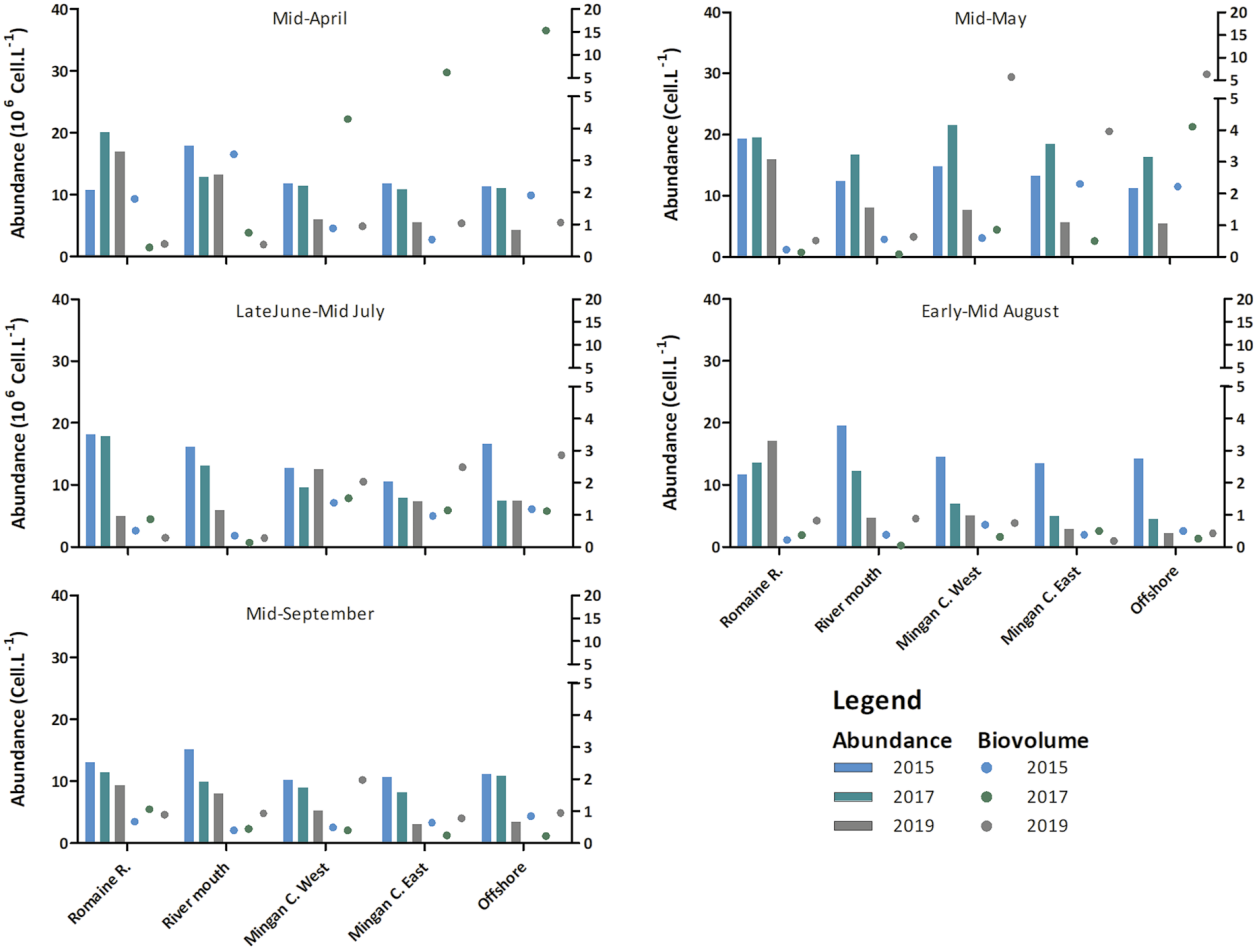
Phytoplankton abundance and biovolume in the study area from April to September, in 2015, 2017 and 2019

To complement this analysis of abundance, trends in total biovolume and total abundance are shown in Fig. 4. The analysis is supported by data on the relative distribution of the biovolumes of the different taxonomic groups considered (Sup.Info.I). In April, the phytoplankton community of the Rivière Romaine (P0) contained between 10.7 10^6^ cells L^−1^ and 20.0 10^6^ cells L^−1^, with a biovolume ranging from 0.3 to 1.8 mm^3^ L^−1^. In 2017 and 2019, the community was clearly formed of numerous smaller cells than the community in the Chenal de Mingan, mostly diatoms and brown algae (50% and 40% of the biovolume respectively, Sup.Info.I). In May, flagellates and chlorophytes replaced brown algae, and the community was at its most abundant overall, with an average of 18.2 10^6^ cells L^−1^, resulting in the lowest biovolume across years and seasons was recorded in this month (0.3 mm^3^ L^−1^). In the SFP, the waters warmed up, and flagellates replaced diatoms to dominate the community. The biovolume then increased, ranging from 0.2 to 1.6 mm^3^ L^−1^, while abundance declined, with highest values recorded in July (maximum of 18.1 10^6^ cells L^−1^ in 2015) and lowest in September (minimum of 9.4 10^6^ cells L^−1^ in 2019). Offshore, on the other hand, the community was smaller in April, especially in 2019 (4.2 to 11.3 10^6^ cells L^−1^), with a larger biovolume ranging from 1.1 to 15.3 mm^3^ L^−1^. The presence in the area of the large diatom *Azpeitia tabularis* (Coscinodiscophyceae) is responsible for that maximum value observed in 2017. Without this class of diatoms, the biovolume would have been 3.9 mm^3^ L^−1^ in 2017, still higher than those recorded in 2015 and 2019. Overall, with or without the Coscinodiscophyceae, diatoms dominated the community in April. The population grew from April to May, and though no *A. tabularis* were found, biovolumes were still high, averaging 4.2 mm^3^ L^−1^. Another particularity of 2017 was the lower relative importance of diatoms compared to flagellates in May: while diatoms dominated the community in terms of biovolume in 2015 and 2019 (> 90%; Sup.Info.I), they accounted for only 54% of the offshore community in 2017, flagellates accounting for the other 46%. In all three study years, offshore abundance and biovolume decreased from July to August, followed by an increase in abundance in September in 2017 and 2019. Diatoms still dominated in late June/early July, but after that flagellates, green algae and cyanobacteria were abundant enough to account for a significant (21% in 2017) to major portion (77% and 80% in 2015 and 2019) of the biovolume. The variations in abundance vs. biovolume in September again reflected the succession patterns encountered over the study years: a shift towards a relatively small community composed of large diatom cells was noted in 2019, while 2017 saw a large and diverse community of small biovolume, with specimens of all targeted phytoplankton groups (Fig. 4 and SI1).

Abundance, biovolumes and taxonomic distribution in the intermediate stations reflect the influence of freshwater on the estuary and its limitations. At station P3, directly at the river mouth, abundances were similar to those in the river, but it is the succession pattern of the taxonomic groups (according to biovolume, SI1) that clearly demonstrates the strong river signature in all periods across the years. Nonetheless, unexpected differences in the succession pattern at this station (a strong increase in green algae in August 2019, for example) demonstrate the particularity of the dynamic environment of the mixing zone. Farther from the coastline, in the eastern and western parts of Chenal de Mingan, the trends in abundance, biovolume and taxonomic succession are clearly patterned on those of the offshore station.

### Zooplankton dynamics

**Fig. 5:**
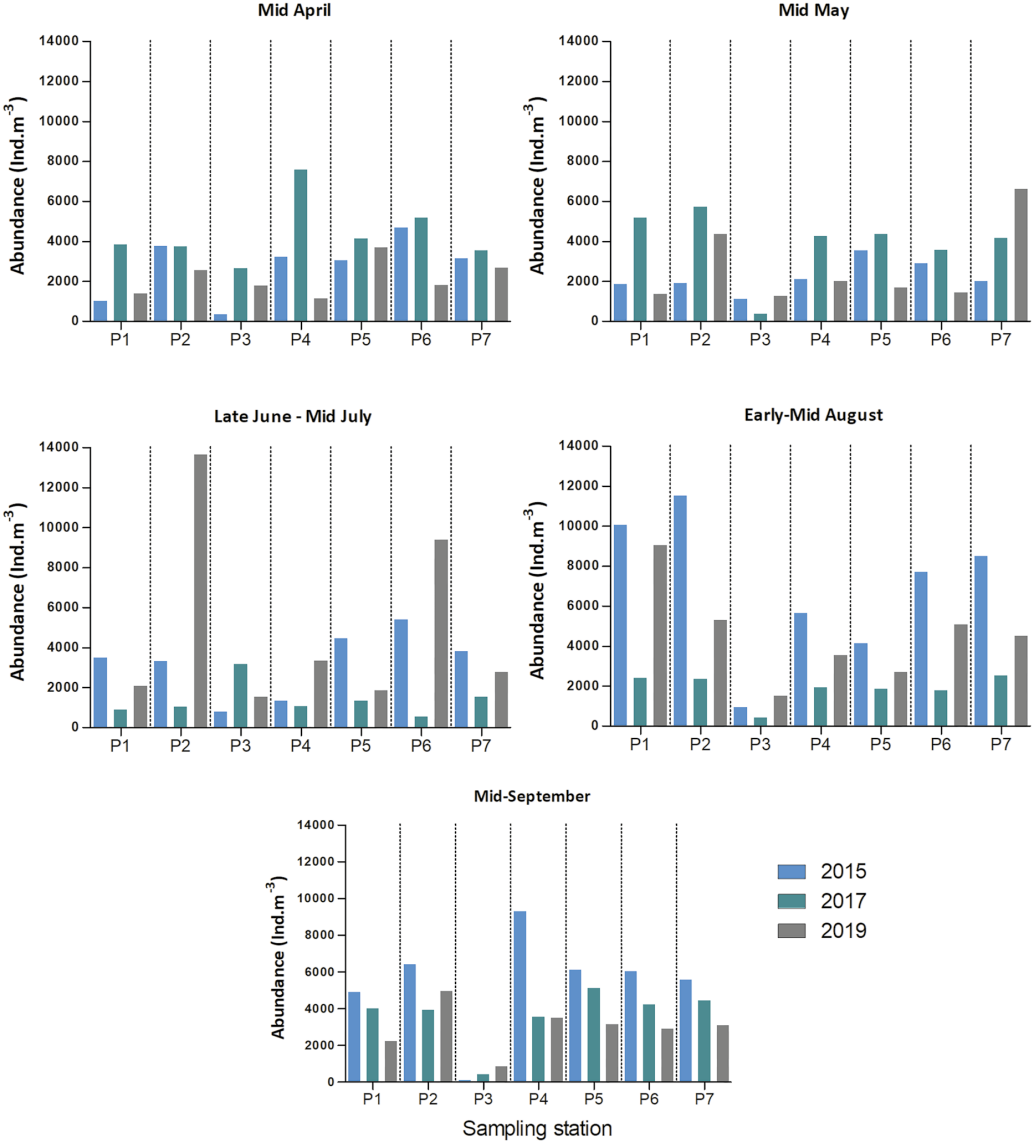
Zooplankton abundance (ind m^−3^) at sampling stations P1 to P7 in 2015, 2017 and 2019

Zooplankton were sampled at the river mouth (station P3) and in the Chenal de Mingan. Analyses showed the distribution of the different zooplankton groups to be similar at the stations in the Chenal de Mingan but different from that at station P3. Overall, zooplankton abundance ranged from 114 to 13 333 ind m^−3^ (Fig. 5) and was significantly lower at P3 (1 154 ind m^−3^ in average; SD ± 878) compared to offshore stations (3 945 ind m^−3^ in average; SD ± 2 448 Student’s *T*-test *p* < 0.05). In 2015 and 2019, abundance was slightly lower in April and May than in other months (ANOVA, *p* > 0.05), but this weak pattern was not observed in 2017 because of the low summer values. In July 2019, abundances at P2 and, to a lesser extent, at P6 were higher, at 13 666 and 9 397 ind m^−3^ respectively. These numbers were inflated, however, by the presence of jellyfish, echinoderm and gastropod larvae (> 5 000 ind m^−3^), organisms that in adulthood are no longer classified as zooplankton. The high values reported should accordingly be treated with caution, since they do not represent a zooplankton growth peak but rather increased recruitment of higher organisms. Removing these organisms from the comparisons yields zooplankton abundances at P2 and P6 comparable those at the other stations.

Overall, the following succession was identified at the offshore stations: in April and May, juvenile zooplankton organisms (nauplii) were dominant, and in July, August and September, a substantial number of larvae (jellyfish and starfish) were captured. Cyclopoids and calanoids dominated, each group accounting for between 11 and 61% of the community Sup.Inf.II)). At the river mouth (station P3), the succession was different because of the major dominance of cladocerans. Nauplii accounted for 49% and 72% of the population in April 2019 and 2017, respectively. In the following months of 2017, calanoids first dominated and were then replaced by cladocerans, which accounted for 60% of the community in September. A similar pattern was noted in 2019, except that many gammaria and larvae were captured in July. In early spring 2015, few nauplii were found at the river mouth and the community was already dominated by cladocerans. Nauplii were more abundant from May to September, and the community was also more diverse and balanced with four to five groups accounting for 7 to 50% of the community.

## Discussion

### Water quality parameters in the Chenal de Mingan unaffected by modulation of the Rivière Romaine flow rate

Surface water temperature and salinity in the study area are altered in complex ways in response to changes in dynamic factors, i.e. tidal cycle, river discharge and distance from the river mouth. In every study year, there were differences between surface water temperatures measured at the two instrumented buoys, mainly due to their positions, regardless of the season and associated variations in air temperature. Station P1 is near the coast, in a shallow area influenced by surface waters from the Lechasseur and Romaine rivers, i.e. freshwater exports that change and warm up in summer. Station P2 is located directly in the Chenal de Mingan, where temperature and salinity are likely influenced by cold offshore water from the Détroit de Jacques-Cartier (Senneville et al., 2018). The tidal cycle also modulates surface salinity and temperature at both stations. At flow tide and high tide, offshore waters are pushed towards the coast, confining freshwater dispersion to the mouth of the Rivière Romaine (Demarty et al., 2020). Under these conditions, high salinity is measured at station P1, and station P2 is outside the freshwater influence zone. At ebb and low tides, the tidal front retreats into the Chenal de Mingan, allowing the accumulated freshwater to disperse in the estuary and offshore before being washed away and diluted in the surrounding dominant saline waterbody. Under these conditions, low salinity is measured at station P1 and all the way to station P2. Lastly, changes in discharge from the Rivière Romaine have a cumulative effect on the tidal cycle. Dispersion potential is high when discharge is high but lower under low discharge conditions. Such changes in discharge conditions coupled with the tidal cycle modulate salinity and temperature in the estuary. An example of such modulation was noted during the first years of operation of the Romaine complex, especially during the HFP of 2017 (low discharge) and the SFPs of 2015 and 2017 (high discharge), resulting, respectively, in higher salinities in the estuary during freshet and lower salinities during the SFP (Senneville et al., 2018; Demarty et al., 2019; Deblois et al., 2019). The 2019 hydrograph was closer to baseline conditions than the hydrographs of 2015 and 2017. The discharge conditions in 2019 combined with the tidal cycle induced variations in salinity very similar to those observed under natural conditions in 2013, i.e. lower and variable salinity during the HFP and higher and more stable salinity in the SFP.

Regional factors also modulate water temperature in the Chenal de Mingan. In summer, when the Rivière Romaine footprint is low due to the low discharge, the influence of offshore waters relative to freshwater exports increases. Summer data show surface water temperatures were lower in 2013 and 2017 than in 2015 and 2019. Compared to the reference, freshwater exports were 22.3% greater in 2015 and 4.3% smaller in 2019. Yet both of these conditions resulted in an increase in water temperature in the study area. Thus, variations in water temperature in the study area cannot be explained solely by changes in the interannual hydrological characteristics of the Rivière Romaine or distance to shore. In fact, according to data from the Atlantic Zone Monitoring Program (AZMP) (Galbraith et al., 2020), surface water temperatures near the coast (Chenal d’Anticosti) were 1 to 3 °C higher in 2015 and 2019 than historical temperatures, especially in July and August. Interestingly, in August 2015 and 2019, high-tide temperatures at stations P1 and P2 were higher than usual compared to low-tide temperatures, demonstrating that the offshore waters were warmer (Demarty et al., 2021). In light of the AZMP results, the higher surface water temperatures in August make sense and can be attributed to the warmer offshore waters brought into the study area at high tide. This finding suggests that changes in regional temperatures may be responsible for some of the variations noted in the study area and that modulation of the Rivière Romaine is not the only factor at work in determining conditions in the Chenal de Mingan. This finding also underlines the importance of considering climate variability and regional trends linked to climate changes when analyzing a project’s impact on the environment, and it also highlights the importance of maintaining reliable meteorological and limnological networks to support regional studies (Ely et al., 2020).

In addition to possible impacts of changes in freshwater flow rate on physical characteristics at the river mouth, one of the main concerns linked to reservoir creation is the change in nutrient loading and its impact on the estuary foodweb. While freshwater from the Boreal region is generally considered phosphorus (P) limited, coastal and marine environments are considered nitrogen (N) limited, and this is true of the Golfe du Saint-Laurent (Blais et al., 2019; Howarth & Marino, 2006; Schindler, 1977; Wetzel, 2001). When terrestrial ecosystems are flooded to create reservoirs, allochthonous carbon and nutrients are released to the water column, enhancing bacterial productivity and stimulating production overall in the reservoir ecosystem (trophic upsurge, Kalff, 2002). In the northeastern Boreal region of Québec, however, the trophic upsurge is limited, and reservoirs remain oligotrophic, like natural lakes in the area, even in the early years after first flooding (Bogard & Del Giorgio, 2016; Schetagne, 1994). Our results show interannual differences in nitrate concentrations measured in the Rivière Romaine after flooding, but these remained within the range of differences measured under natural conditions (Hydro-Québec, 2007) and those expected in rivers in the region (Wetzel, 2001). Low dissolved ammonium concentrations also indicate that the river does not contribute significantly to nitrogen loading in the waters of the Chenal de Mingan. Like TDN, phosphorus concentrations in the Rivière Romaine were low and characteristic of oligotrophic Boreal freshwater throughout the study period (Hydro-Québec, 2007; Wetzel, 2001). However, phosphorus concentrations were two to five times higher in the Chenal de Mingan, and phosphorus and nitrate concentrations strongly covaried in this area, as throughout the Golfe du Saint-Laurent (Blais et al., 2019). While nitrogen and phosphorus are essential for the growth of all primary producers, reactive silica is a particular nutrient in that it is essential for the growth of diatoms (Wetzel, 2001). Diatoms dominate the algal community biomass of the Golfe du Saint-Laurent, especially in cold water during the spring bloom. Silica concentrations recorded in the river and in the Chenal de Mingan in our study remained within the range of variation observed throughout the Golfe du Saint-Laurent (Blais et al., 2019).

Besides finding that nutrient load did not vary significantly from one year to the next and that concentrations in the estuary and in the Chenal de Mingan did not covary with discharge rate or nutrient concentrations in the Rivière Romaine, our study’s major contribution is the demonstration that the impoundment of three reservoirs on the Boreal Rivière Romaine had no measurable impact on nutrient stoichiometry in the Chenal de Mingan. In fact, increasing the load of a non-limiting nutrient or, conversely, decreasing input of a limiting nutrient can have a limited impact on the receiving ecosystem. On the other hand, a change in nutrient stoichiometry in favour of a limiting nutrient can have a devastating impact on an aquatic environment (Maarava et al., 2020). Redfield (1963) established that to support the growth of phytoplankton the optimum nutrient ratio of nitrogen to phosphorus (N:P) is 7.2 (Redfield ratio, molecular weight basis). In the Rivière Romaine, the N:P ratio was approximately 20, i.e. the river is phosphorus limited, as expected in freshwater. The N:P ratio in the Chenal de Mingan, on the other hand, never exceeded 4.1, confirming the nitrogen limiting conditions in the Golfe du Saint-Laurent as described in the literature (Blais et al., 2019). Silica is also important in this ecosystem because of the prevalence of diatoms. There is no Si:N ratio as well-established as the Redfield N:P ratio. The literature reports ratios ranging from 1 to 8 (molecular weight basis) for the northwest European shelf seas, which are dominated by diatoms during the spring bloom (Tett et al., 2003). In the Rivière Romaine and the Chenal de Mingan, ratios were approximately 5 regardless of the Rivière Romaine discharge rate. Considering a non-limiting Si:N ratio of 1, this suggests silica was not limiting for diatoms throughout the study area. Furthermore, TDN was high every year in early spring and invariably dropped with the onset of the phytoplankton spring bloom, confirming that nitrogen availability is a key element for phytoplankton in this ecosystem. In other words, anthropogenic changes to the river due to construction and operation of the Romaine complex did not cause significant modification of the nutrient load or, more importantly, of the controlling nutrient at the river mouth and thus should not alter the planktonic community in the Chenal de Mingan.

### Variability of the coastal planktonic community driven by the Golfe du Saint-Laurent

Our findings demonstrate the very limited impact of the Rivière Romaine on physical and chemical parameters in the Chenal de Mingan. Statistical analyses showed no relationship between chlorophyll *a* concentrations in the study area and either nutrient loading from the river or the river’s discharge rate. To our knowledge, the plankton community succession described herein constitutes the first exhaustive, multiyear study of the rich natural environment of the Archipel de Mingan.

In the Chenal de Mingan, chlorophyll *a* concentrations overall ranged from 0.1 to 7.6 µg L^−1^, i.e. within the expected range (0–15 µg L^−1^) for the Golfe du Saint-Laurent (Blais et al., 2019). The increase/decrease in chlorophyll *a* concentrations noted in April/May in the time series (Fig. 3) correlates with a marked decrease in nitrate concentrations in the water column. This concordance reflects the assimilation of nutrients during flowering by primary producers dominated by diatoms, as anticipated in the EIS for the Romaine complex project (Hydro-Québec, 2007). Diatoms are well-adapted to cold oligotrophic water that is not too turbid and is rich in silica, like the waters of the Chenal de Mingan (Bérard-Therriault et al., 1999; Blais et al., 2019; Le Fouest et al., 2005; Sommer, 1994). During the summer, phytoplankton growth was not only limited by nitrate availability (bottom control) but also under predation pressure (top control). These dynamics caused cycles of rising and falling growth during the SFP that are clearly visible in the chlorophyll *a* time series of the buoys (Fig. 3). In 2015, these cycles were visible in July and August and accompanied by a drop in nitrates in the water column as well as a spike in zooplankton abundance. In 2017 and 2019, this concordance was not as obvious, probably because of a mismatch in the timing of the sampling campaigns, but the presence of three chlorophyll *a* peaks shows the plankton growth cycles were still present in the water column.

Zooplankton develop in bursts of growth which usually begin shortly after the phytoplankton growth episodes and stop when phytoplankton become less abundant due to zooplankton grazing. Cyclic growth must be taken into account when interpreting the study results obtained since 2015. In any given campaign, sample collection is sometimes synchronous with a zooplankton growth peak. This was the case, for example, in July 2019, when there was a spike in gammaria growth at the river mouth. In this context, observed differences must be interpreted with care, since some variations are the random effect of synchronization of the sampling period and a peak in growth of zooplankton or the larvae of higher organisms.

In general, our data show that the zooplankton community of the Chenal de Mingan has been composed of the same groups of species since 2015. Abundant at the start of the season, nauplii develop with the growing season into different species of copepods (cyclopoids, calanoids and harpacticoids), with cyclopoids and calanoids tending to dominate until the end of the study period. These groups are in fact usually dominant in the waters of the North Atlantic and the Golfe du Saint-Laurent (Blais et al., 2019). In 2017 and 2019, gastropods, bivalves, echinoderms and tunicate larvae were also abundant in the study area, their presence in the zooplankton net throughout the growing season demonstrating recruitment of these species. Regardless of the river discharge, the community at the mouth of the Rivière Romaine was dominated by cladocerans, which are usually prevalent in freshwater. These organisms may have been abundant throughout the river continuum and have been carried by the river to the mouth, remaining confined there where salinity is relatively low.

## Conclusion

This study paints the following portrait of the impact of the Romaine complex on plankton production. At its estuary, the Rivière Romaine becomes a mass of surface freshwater whose extent and salinity depend on the modulations in river flow. Since the first commissioning of the Romaine complex, in 2015, modulations in river flow (target period, duration and flood flow) have resulted mainly in changes in variations in surface salinity at the mouth, specifically north of Île de la Grosse Romaine, where the freshwater seems confined. In the Chenal de Mingan, however, water column salinity and temperature are not affected by the river water discharge as postulated in our work hypothesis but are mainly affected by the mass of water coming from the Détroit de Jacques-Cartier, i.e. the Golfe du Saint-Laurent. Our results also show that nutrient contribution and planktonic biomass from the Rivière Romaine are not significant factors and that, on the contrary, there is a clear relationship between nutrient content at the river mouth and what come in at high tide with the open sea. Based on the collected data, our work hypothesis is rejected and we can conclude that modulation of the discharge rate of the Rivière Romaine and the associated changes in water quality have not generated measurable changes in plankton production in the Chenal de Mingan.

## Data Availability

The datasets generated and/or analyzed in this study are available from Hydro-Quebec on reasonable request (centredoc@hydro.qc.ca).
